# Amyloid-β modulates the phase separation and aggregation of α-synuclein

**DOI:** 10.1073/pnas.2501987122

**Published:** 2025-07-14

**Authors:** Alexander Röntgen, Zenon Toprakcioglu, Owen M. Morris, Michele Vendruscolo

**Affiliations:** ^a^Centre for Misfolding Diseases, Yusuf Hamied Department of Chemistry, University of Cambridge, Cambridge CB2 1EW, United Kingdom

**Keywords:** protein aggregation, protein condensation, liquid-liquid phase separation, chemical kinetics

## Abstract

The aggregation of amyloid-β (Aβ) and α-synuclein (αSyn) into insoluble proteinaceous deposits is a hallmark of Alzheimer’s and Parkinson’s diseases. Recent evidence suggests that these amyloidogenic proteins act in synergy, with their coaggregation frequently observed in these disorders. In this study, we investigate the interaction of Aβ and αSyn using various biophysical tools. In particular, we explore the cocondensation of Aβ with αSyn, elucidating the pathways through which Aβ modulates αSyn phase separation. We studied different variants of Aβ, focusing on the most prominent species, namely Aβ42 and Aβ40. We found that Aβ42 and Aβ40 have fundamentally different mechanistic effects on the kinetics of αSyn condensation. Aβ42 initially forms large aggregates that act as heterogeneous nucleation sites which initiate the phase separation of αSyn. In contrast, Aβ40 is sequestered into αSyn condensates where it accelerates the liquid-to-solid transition of αSyn into amyloid aggregates. All other Aβ variants we probed fell into one of these two mechanistic pathways, with Aβ37, Aβ39, and Aβ35-25 exhibiting similar behavior to Aβ40, whereas Aβ43 triggered nucleation processes similar to Aβ42. Given the complexity behind amyloid formation, it is key to understand how molecular partners can interact with one another. Our results thus illustrate the extreme sensitivity of protein mixtures and shed light on some of the mechanisms involved in the cocondensation and aggregation of Aβ with αSyn.

## Significance

The coaggregation of amyloid-β (Aβ) and α-synuclein (αSyn) has been implicated in Alzheimer’s and Parkinson’s diseases, yet the molecular mechanisms behind these processes remain elusive. By investigating the cocondensation of Aβ with αSyn, we reveal that Aβ42 and Aβ40 influence αSyn phase separation through distinct kinetic pathways. Aβ42 forms early aggregates that act as nucleation sites for αSyn phase separation, while Aβ40 is sequestered into αSyn condensates, accelerating the liquid-to-solid transition. Additionally, the Aβ isoforms Aβ37, Aβ39, and Aβ35-25 displayed similar behavior to Aβ40, while Aβ43 followed the same pathway as Aβ42. These findings offer insights into protein cocondensation and aggregation mechanisms regarding neurodegeneration. Through such investigations, new avenues for therapeutic targeting of Aβ and αSyn cocondensation can be explored.

Alzheimer’s disease (AD) and Parkinson’s disease (PD) are neurodegenerative disorders characterized by a progressive loss of neuronal function (1–3). These conditions are closely associated with protein misfolding and aggregation (4, 5), a process that leads to the formation of pathological inclusions of amyloid-β (Aβ) in AD (1, 3) and α-synuclein (αSyn) in PD (2, 6). The association of these deposits with disease, however, is still incompletely understood. Following initial reports of the presence of αSyn in amyloid plaques (7, 8), the phenomenon of protein coaggregation has been increasingly investigated to reveal its possible roles in disease comorbidity (9–13). In parallel, the effects of aggregating proteoforms, including mutational variants (1–3), splice isoforms (14, 15), and posttranslational modifications (1–3), have also been investigated. Therefore, understanding the role of protein aggregation in neurodegeneration and its translation for the development of diagnostic and therapeutic approaches is complicated due to the plethora of mechanisms which govern the above processes.

Further complexity is added by the recent discovery of liquid–liquid phase separation as an alternative pathway for the self-assembly of biomacromolecules, which has been extensively researched in the past years (16–20). Given the disordered nature of the liquid condensates, various proteins are prone to undergoing cocondensation and this process is believed to be the basis of the formation of membraneless organelles in the cell (16, 21). The concentration of amyloidogenic proteins into liquid condensates facilitates their nucleation into the amyloid state. Consequently, the formation of amyloid aggregates within liquid-like intermediate condensates has been reported for numerous proteins including αSyn, Aβ, tau, fused in sarcoma (FUS), and transactive response DNA binding protein 43 (TDP-43) (22–26).

For αSyn, there is an increasing number of studies investigating external factors that may modulate its phase separation and subsequent aggregation within condensates, including salts, metal ions, lipids, aliphatic alcohols, and other small organic molecules (23, 24, 27, 28). Moreover, given the vast spectrum of biomolecules involved in cellular function, it is quite plausible that, following condensate formation, one protein may interact and alter the phase behavior of another.

As both αSyn (23, 24, 29) and Aβ (30) have been shown to independently phase-separate, it is important to explore their interplay under cocondensation settings. A potential pathway for the interaction between Aβ42 and αSyn is that extracellular Aβ is initially internalized via endocytosis, which can result in the intracellular concentration of Aβ42 being enhanced into the low micromolar range (31–33). However, to date, the phase behavior of systems composed of mixtures of Aβ and αSyn remains poorly understood, with previous studies focusing on protein aggregation and not phase separation (34–37). One possibility is that the initial formation of aggregates of one protein may catalyze the formation of aggregates of the other protein by heterogeneous nucleation (35). However, the way in which these proteins interact with one another in the condensed phase remains unclear.

To address this question and explore it in greater detail, in this work, we investigated the interplay between Aβ and αSyn as they cophase-separate. We studied how the two most common variants of Aβ, Aβ42 and Aβ40, elicit different effects on the phase behavior of αSyn. We discovered distinct pathways through which Aβ influences the kinetics of αSyn condensation. We found that Aβ42 first forms aggregates, which in turn nucleate the condensation of αSyn. On the other hand, Aβ40 remains soluble at first, is recruited into αSyn condensates, and thereby facilitates the aggregation of αSyn. Importantly, other Aβ variants follow one of these two pathways, with Aβ37, Aβ39, and Aβ35-25 exhibiting similar characteristics as Aβ40, while the behavior of Aβ43 resembled that of Aβ42. Our results point out the significance of exploring the interactions between Aβ and αSyn, especially in the context of understanding the possible mechanisms of cocondensation underlying neurodegenerative diseases.

## Results

### Aβ42 Colocalizes within αSyn Condensates.

We first investigated the propensity of Aβ42 to interact with αSyn condensates using a combination of biophysical techniques, chemical kinetics, and fluorescence microscopy (23). To explore how Aβ42 modulates αSyn phase separation, we deposited a small volume of a mixture containing αSyn and Aβ42 onto a glass microscope dish and monitored the process over time. By employing confocal microscopy, we were able to study the effect of Aβ42 on the biophysical properties of the αSyn liquid condensates. More information regarding the experimental protocol can be found in the Methods section. The samples contained a fixed concentration of αSyn (100 μM) with varying concentrations of Aβ42. Moreover, each sample contained a small proportion of labeled αSyn and Aβ42 (AF647-αSyn and AF488-Aβ42) to visualize the phase separation of each of the proteins.

**Fig. 1.**
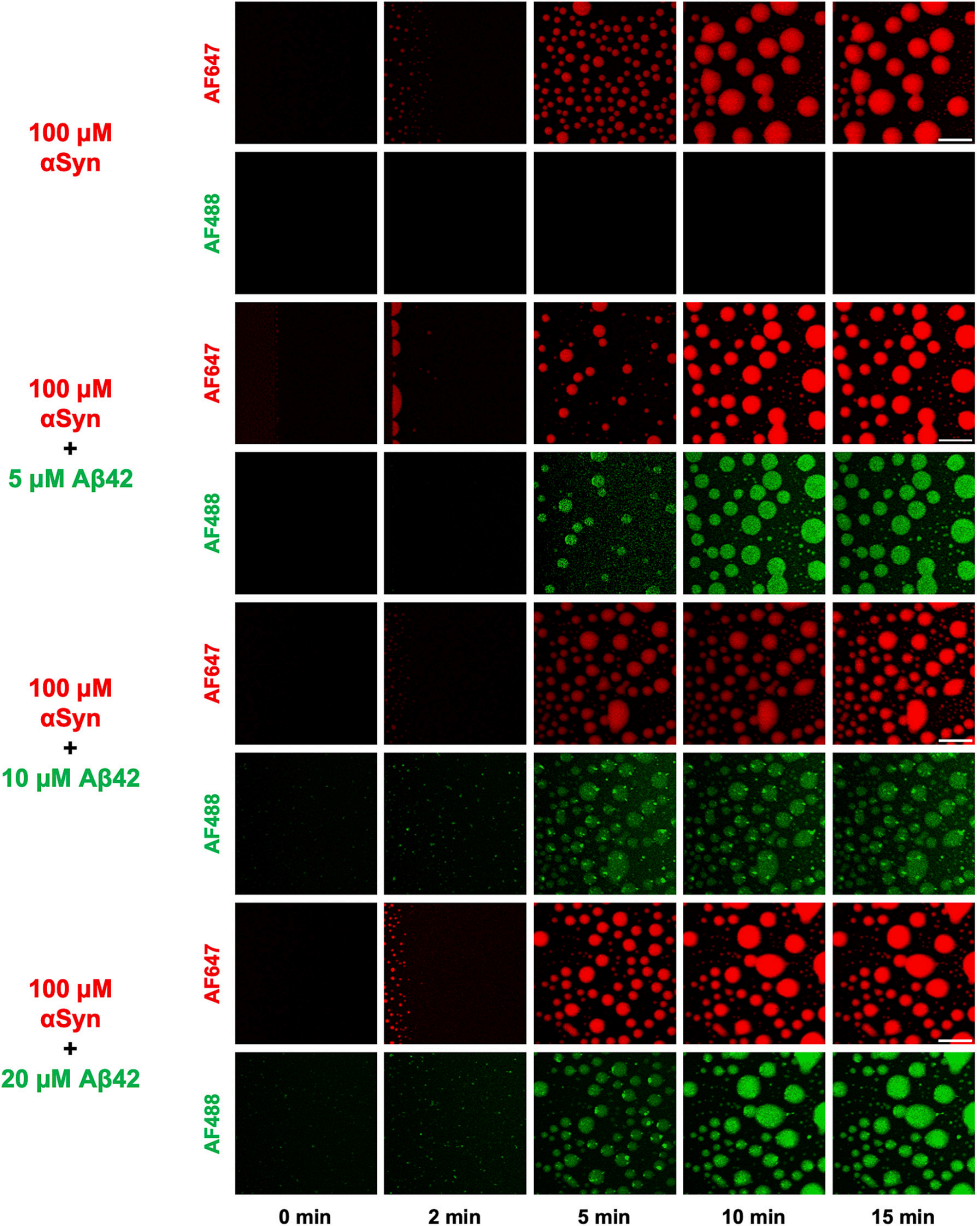
Aβ42 colocalizes within αSyn condensates. Time-lapse confocal microscopy images displaying the liquid–liquid phase separation of αSyn (1% labeled with AF647, shown in red) with increasing amounts of Aβ42 (10% labeled with AF488, shown in green). The colocalization of Aβ42 within the αSyn condensates can be seen for all concentrations of Aβ42 tested. (Scale bar, 10 μm.)

We determined that, for all conditions tested, liquid–liquid phase separation was observed within minutes (Fig. 1). Additionally, a control sample composed solely of αSyn underwent liquid–liquid phase separation, with dense droplets exhibiting characteristic coalescence and ripening (Fig. 1, *Top* panels). Over time, these processes resulted in fewer droplets with larger diameters (Fig. 1), which has also been observed previously (23). Conversely, a sample composed entirely of Aβ42 did not undergo liquid–liquid phase separation, but instead aggregated directly within a dilute liquid phase (*SI Appendix*, Fig. S1). Interestingly, when mixing αSyn with Aβ42, small clusters of Aβ42 are present in the αSyn condensates. This is more evident when higher concentrations of Aβ42 were used (i.e., 10 and 20 μM), as shown in the bottom panels of Fig. 1.

### Aβ42 Aggregates Promote αSyn Phase Separation.

**Fig. 2.**
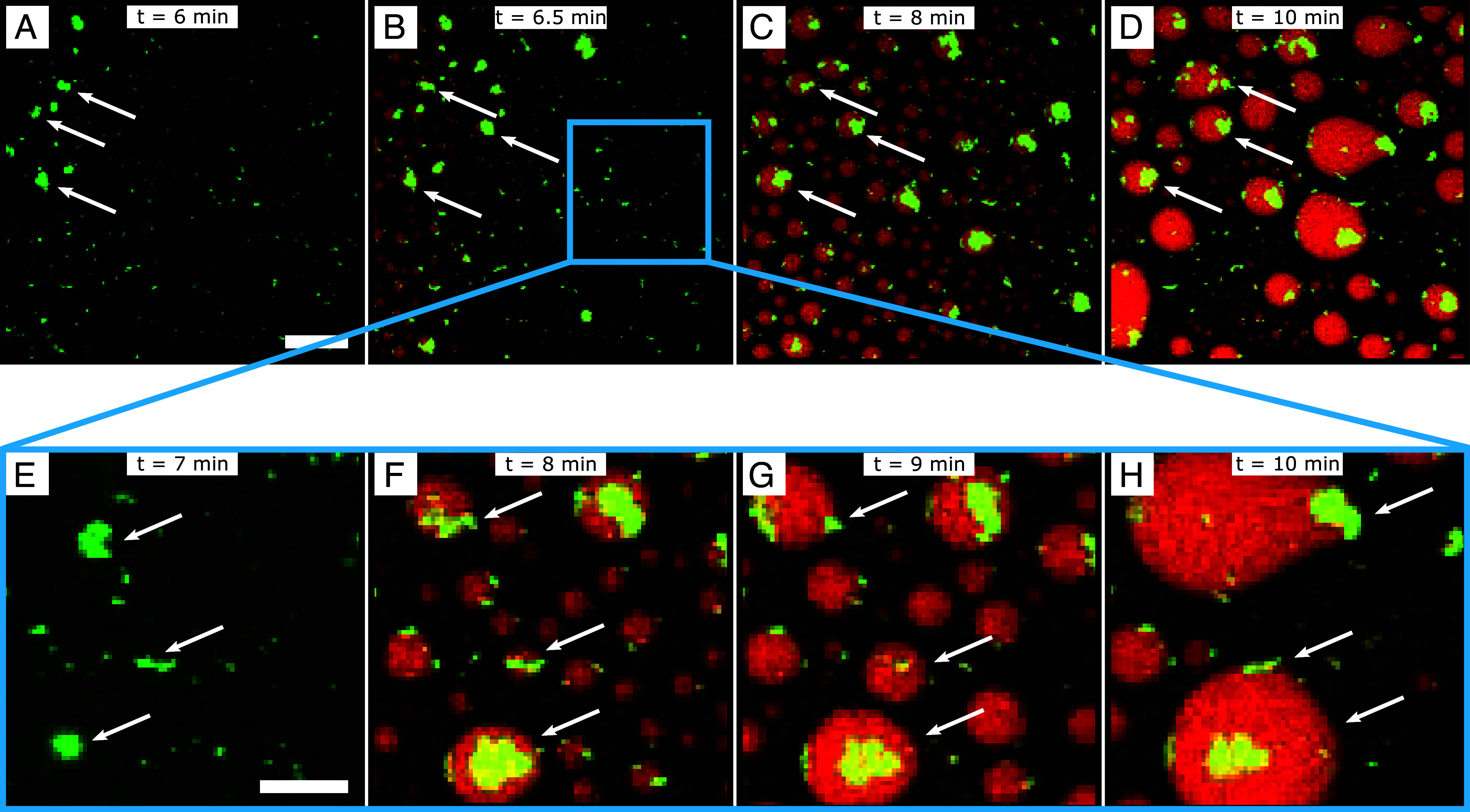
Aβ42 aggregates trigger the phase separation of αSyn. (*A*–*D*) Time-lapse confocal microscopy images showing how Aβ42 (green) first aggregates (*A*) before acting as a nucleation site from which αSyn condensates (red) form (*B*). (*C*) Subsequent αSyn condensates are formed over the Aβ42 aggregates. (*D*) The αSyn droplets coalesce and grow until they too aggregate forming solid structures. The white arrows show the initial Aβ42 aggregates and how subsequent αSyn condensates form over them. (Scale bar, 10 μm.) (*E*–*H*) Magnified time-lapse confocal microscopy images from panel (*B*). The mechanism through which Aβ42 aggregates trigger αSyn phase separation can be seen in greater detail. (Scale bar, 5 μm.)

To explore the effect of Aβ42 aggregates colocalizing with αSyn condensates in greater detail, we focused on the mixture of 10 μM Aβ42 with 100 μM αSyn. Using fluorescence microscopy, we found that Aβ42 initially forms aggregates (shown in green in Fig. 2). This process is indicated by the white arrows in Fig. 2*A*. As time progresses, αSyn condensates (shown in red) start forming around the Aβ42 aggregates (Fig. 2*B*). Tracing these specific condensates (indicated by the white arrows) illustrates their further growth and coalescence over time (Fig. 2 *C* and *D*). In fact, all αSyn condensates appear to nucleate over Aβ42 aggregates (Fig. 2*C*). This is further shown in Movie S1. Moreover, this process is again demonstrated in greater detail using higher magnification in Fig. 2 *E*–*H*. Hence, in this time-course sequence, we observe that Aβ42 aggregates trigger the liquid–liquid phase separation of αSyn (Fig. 2 *E*–*F*), and the αSyn condensates are still liquid as they grow and coalesce (Fig. 2 *F*–*H*). Interestingly, the Aβ42 aggregates reside with the αSyn condensates and are able to migrate along with the αSyn condensates when there are coalescence events (bottom two condensates shown in Fig. 2 *G* and *H*). This observation suggests that, while the Aβ42 aggregates are solid, they remain with the αSyn condensates and do not inhibit αSyn phase behavior. Therefore, it appears that Aβ42 modulates αSyn phase separation by acting merely as an anchor point through which phase separation initiates.

### Investigating the Effect of Aβ42 on the Aggregation of αSyn Condensates.

**Fig. 3.**
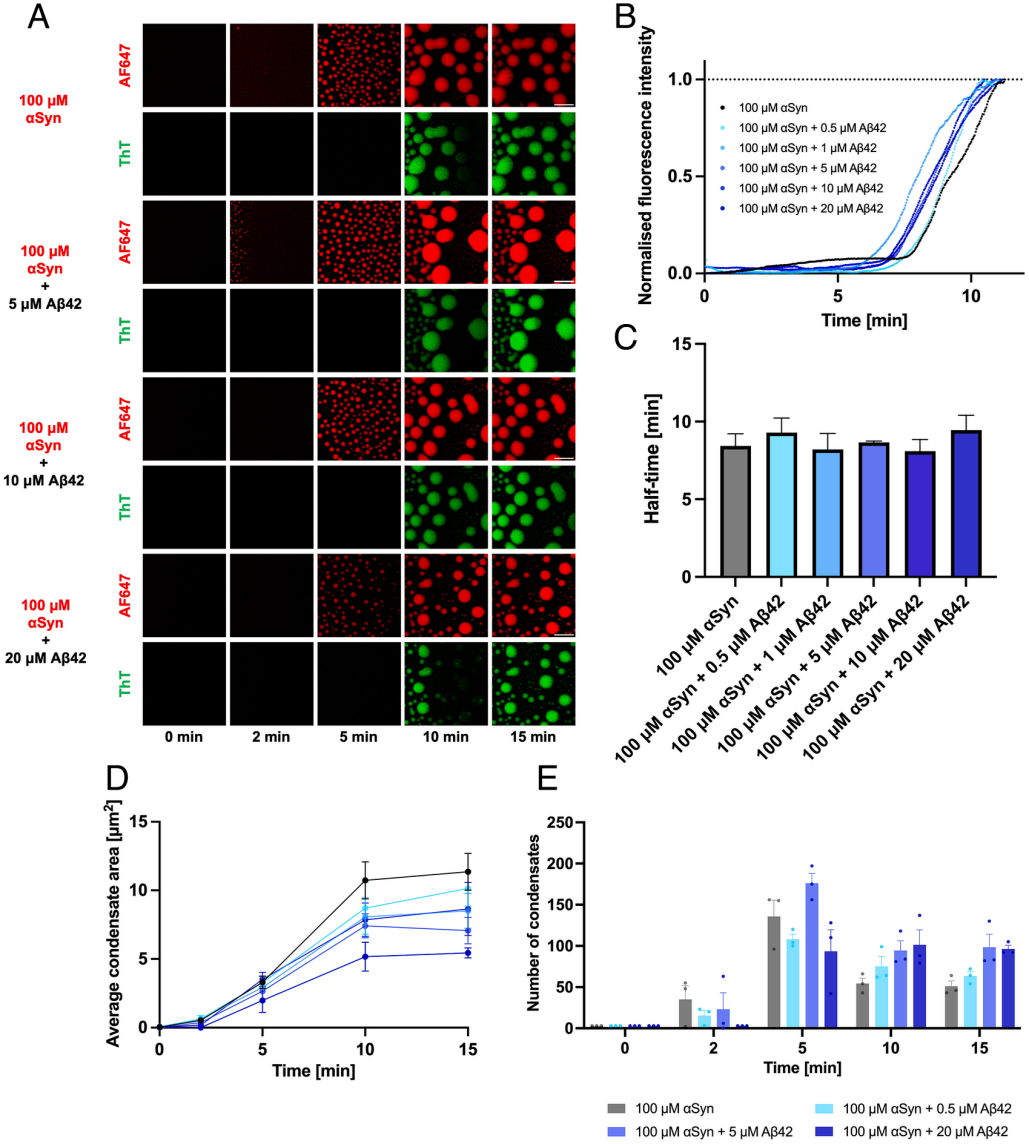
Biophysical analysis of the colocalization of Aβ42 with αSyn condensates. (*A*) Time-lapse confocal microscopy images of αSyn condensates (1% labeled with AF647) with increasing concentrations of Aβ42. The ThT channel displays the subsequent aggregation which takes place within the condensates. (Scale bar, 10 μm.) (*B*) Normalized median kinetic traces corresponding to the aggregation of the αSyn condensates, in the presence of increasing concentrations of Aβ42. (*C*) Half-time plots of the corresponding kinetic traces from (*B*). (*D*) Plot showing the average area of αSyn condensates with increasing concentration of Aβ42. (*E*) Plot of the number of αSyn condensates as a function of time, with the addition of increasing amounts of Aβ42.

To further evaluate the effect of Aβ42 on the phase behavior of αSyn, we explored whether Aβ42 may modulate the aggregation of αSyn within the condensates. To study the aggregation of αSyn and Aβ42 within these condensates, we added 20 μM of the amyloid-binding dye ThT. When ThT binds to the β-sheet structure of amyloid fibrils, its quantum yield increases, which results in an increase in its fluorescence signal (38, 39). To eliminate a potential cross-talk between the excitation and emission wavelengths of AF488 and ThT, we only used wild-type (WT) Aβ42 in experiments where we studied protein aggregation via the condensation pathway. In this experiment, liquid–liquid phase separation was observed for all samples, followed by an increase in the ThT intensity at 10 min, which is indicative of protein aggregation (Fig. 3*A*). Importantly, there does not appear to be any obvious concentration-dependent trend, and the behavior of all systems tested was similar.

A time-course of the ThT channel for each condition where Aβ42 was added to αSyn was analyzed. This produced a series of kinetic traces that enabled us to analyze the effect of Aβ42 on the aggregation of αSyn within liquid condensates (Fig. 3*B*). Each of these individual kinetic traces exhibited the typical sigmoidal pattern characteristic of protein aggregation. This is composed of a lag phase where initial nuclei are formed (4). Following this, an exponential growth phase is observed, where the nuclei proliferate by undergoing many elongation and secondary nucleation events (4). Finally, a plateau phase is observed. This occurs when all the protein has fully converted into fibrillar aggregates. Based on these observations, the half-times of protein aggregation within liquid condensates were determined (Fig. 3*C*). The results are in agreement with the confocal microscopy images shown in Fig. 3*A*. The kinetic traces did not show any notable concentration-dependent trends, indicating that Aβ42 does not significantly influence the liquid-to-solid transition of αSyn condensates, but as mentioned above, acts as a nucleation site from which αSyn can phase-separate. Additionally, the half-time values further corroborate that there is no concentration-dependent trend observable with the incremental addition of Aβ42.

After probing the effect of Aβ42 on αSyn condensate aggregation, we investigated the degree to which Aβ42 affected the biophysical processes of the αSyn droplets. This analysis focused on how the area and number of αSyn condensates varied over time as the concentration of Aβ42 increased. For all conditions, we observed notable changes in the average area of the condensates over time. All systems tested exhibited a similar pattern, whereby the average area of condensates increased from 0 to 10 min due to condensate growth processes (Fig. 3*D*). After 10 min, the size of the condensates plateaued for all conditions. This plateau phase was due to the αSyn condensates undergoing a liquid-to-solid phase transition. The time of this liquid-to-solid phase transition is directly correlated with the time of the plateau phase in the median kinetic traces (Fig. 3*B*). Furthermore, a similar trend was observed when the number of condensates was plotted as a function of time (Fig. 3*E*). We found that, as liquid–liquid phase separation progresses, there is an initial increase in the number of condensates from 0 to 5 min. Following this, as the condensates grow and coalesce, there is a reduction in the number of condensates for almost all samples from 5 to 10 min. Beyond 10 min, the number of condensates plateaued, which was found to be true for all Aβ42 concentrations. This phenomenon is due to the liquid-to-solid transition preventing further condensate growth from occurring as the droplets are no longer liquid, resulting in fewer condensates with a larger area.

By further analyzing the confocal microscopy images which displayed the colocalization of Aβ42 into αSyn condensates, we observed that, at Aβ42 concentrations above 5 μM, ThT-positive aggregates form in the dilute phase before liquid–liquid phase-separated droplets are formed (Fig. 3). The presence of ThT-positive Aβ42 aggregates within the αSyn droplets confirms that Aβ42 does in fact aggregate before acting as a nucleation site for αSyn phase separation.

### αSyn Condensates Undergo a Liquid-to-Solid Transition That Initiates from Single Aβ42 Aggregates.

**Fig. 4.**
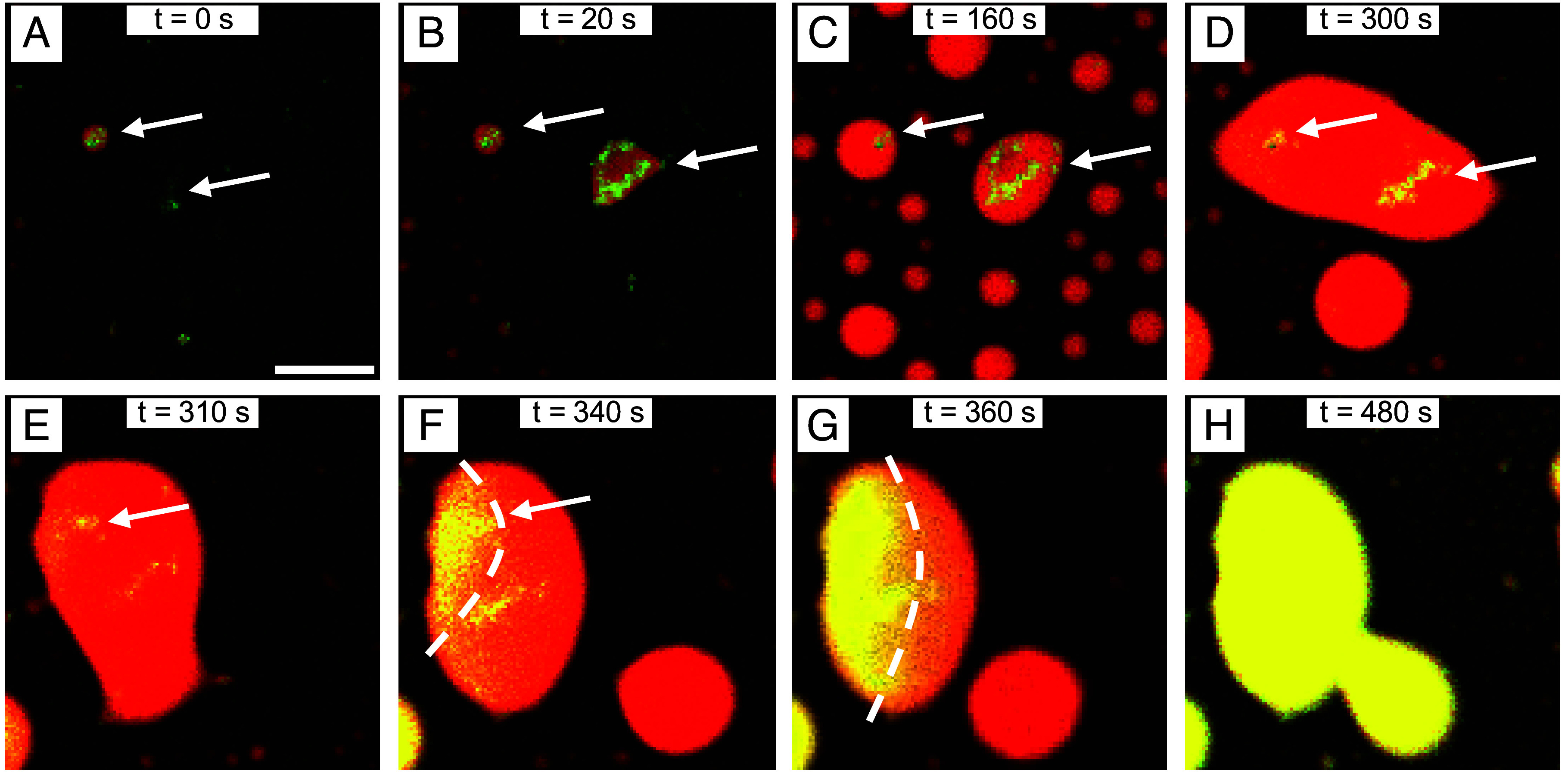
αSyn condensates undergo a liquid-to-solid transition that initiates from single Aβ42 aggregates. (*A*–*D*) Time-lapse confocal microscopy images displaying the initial aggregation of Aβ42 (green), which then nucleate the condensation of αSyn (red). The white arrows show the initial Aβ42 aggregates. These condensates grow and coalesce over time. (*E*–*H*) The condensates undergo a liquid-to-solid transition over time which initiates from a single Aβ42 aggregate (white arrow in panel *E*). The liquid-to-solid transition of the condensate then spatially propagates across the droplet. This is depicted in panels *F* and *G*, where the dotted line displays the wave-like properties of this spatial propagation at different timepoints. (Scale bar, 10 μm.)

We next explored the spatial and temporal evolution behind the liquid-to-solid transition of the αSyn condensates. An individual event illustrating the formation of the condensation of αSyn nucleated by Aβ42, followed by the subsequent maturation and aggregation of the αSyn condensate, is shown in Fig. 4. The image in Fig. 4*A* shows Aβ42 aggregates (shown in green) which subsequently nucleate the condensation of αSyn (Fig. 4 *A*–*D*). The liquid nature of these condensates can be validated by the observation of growth processes. As time progresses, the αSyn condensates begin to aggregate. The aggregation process propagates across the droplet, usually emanating from where a single Aβ42 aggregate is located, thus serving as a discrete nucleation site. This observation suggests that the initial nuclei are critical not only for triggering phase separation but also for controlling the point of origin through which the αSyn condensates will aggregate. The condensate aggregation has a wave-like property and is transmitted throughout the condensates as shown by the dotted line (Fig. 4 *F*–*H*). Finally, the increase of ThT fluorescence intensity (shown in yellow) indicates that the condensate has completely aggregated. The phenomena discussed above are illustrated in Movies S2 and S3. Moreover, we calculated the velocity at which the aggregate wave propagates across the droplet. The velocity of propagation, v_p_, was determined to be 394 ± 53 nm s^−1^, which is in agreement with previous literature values regarding aggregate propagation events in different protein systems (40). Using this velocity, one can then calculate the reaction rate, κ, which describes the overall secondary nucleation processes through which new aggregates are generated from existing ones. Using the equation v = 2√(Dκ), and by assuming the diffusion coefficient to be the same as what has been previously calculated (40), we find that the reaction rate κ = (2.0 ± 0.9) × 10^−2^ s^−1^. This reaction rate is similar to literature values (40), thus suggesting that these protein aggregation mechanisms which propagate from single nuclei follow a similar trend across different protein systems.

### Aβ42 and Aβ40 Elicit Different Effects on the Phase Behavior of αSyn.

**Fig. 5.**
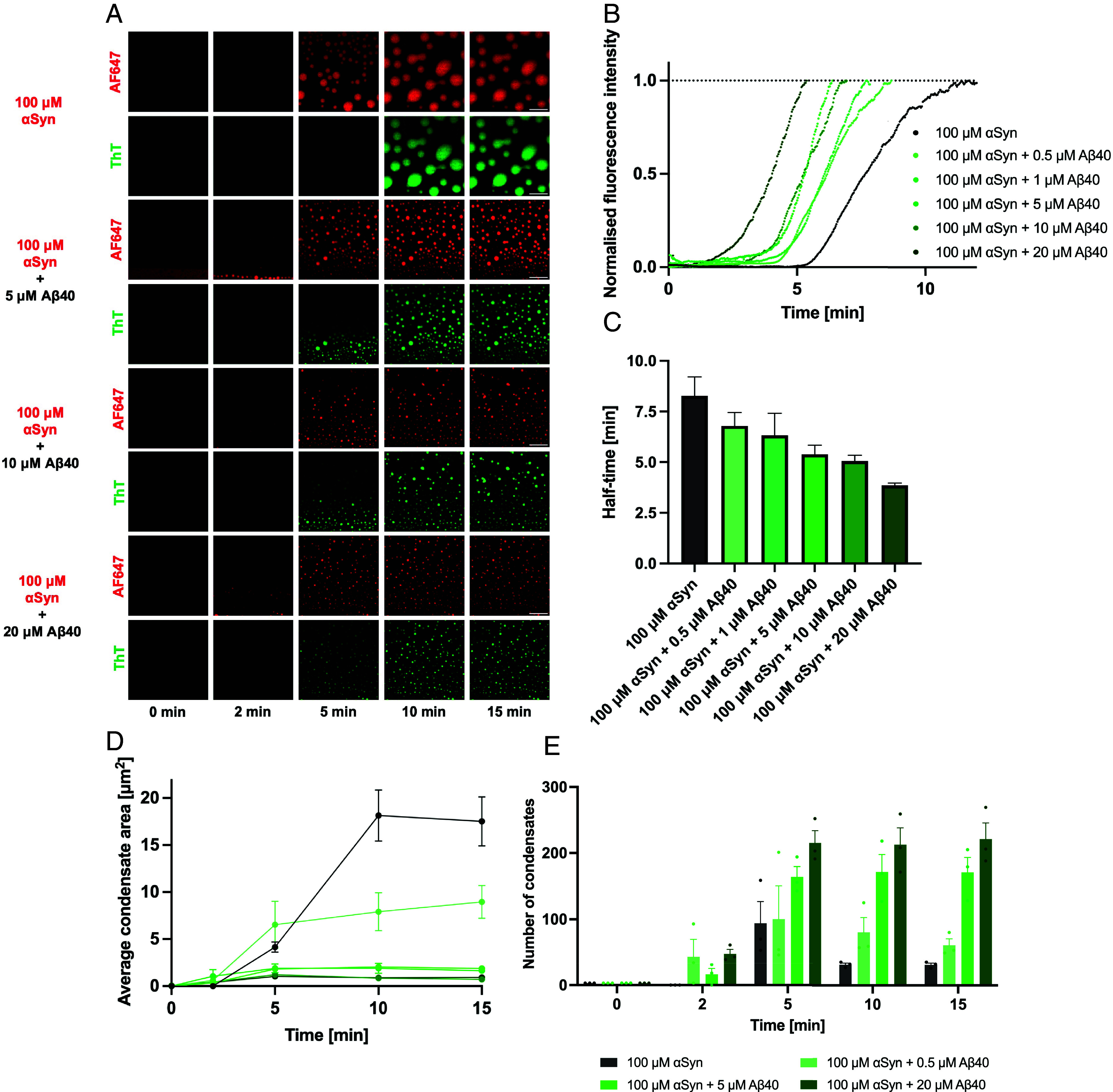
Biophysical analysis of the effect of Aβ40 on αSyn condensates. (*A*) Time-lapse confocal microscopy images of αSyn condensates (1% labeled with AF647) with increasing concentrations of Aβ40. The ThT channel displays the subsequent aggregation which takes place within the condensates. (Scale bar, 10 μm.) (*B*) Normalized median kinetic traces corresponding to the aggregation of αSyn within condensates, in the presence of increasing concentrations of Aβ40. (*C*) Half-time plots of the corresponding kinetic traces from (*B*). (*D*) Plot showing the average area of αSyn condensates with increasing concentration of Aβ40. (*E*) Plot of the number of αSyn condensates as a function of time, with the addition of increasing amounts of Aβ40.

Having established that Aβ42 aggregates can act as nuclei for the phase separation of αSyn, we explored whether another predominant Aβ variant, namely Aβ40, had a similar effect on αSyn. We thus added Aβ40 in a dose-dependent manner to a constant concentration of αSyn (Fig. 5*A* and *SI Appendix*, Fig. S2). We found that Aβ40 is recruited into αSyn condensates (Fig. 5), and the localization of Aβ40 within the condensates was more homogeneous when compared to the localization of Aβ42 (Fig. 1). Moreover, we added 20 μM of ThT in order to determine the effect of Aβ40 on the aggregation of the αSyn condensates. We found that an increase in the concentration of Aβ40 resulted in a corresponding increase in the rate at which the αSyn condensates aggregated. This is shown in the kinetic traces in Fig. 5*B*. The kinetic traces in Fig. 5*B* are derived by measuring the fluorescence intensity values of the ThT channel from Fig. 5*A*. The data are then normalized and plotted as a function of time, so that a direct comparison between the kinetics can be made in a systematic manner. Furthermore, we determined the half-time of aggregation of these kinetic curves, which further corroborates that a higher concentration of Aβ40 resulted in an acceleration of αSyn aggregation (Fig. 5*C*). We believe that this enhancement in the kinetics of αSyn aggregation was due to monomeric Aβ40 interacting with αSyn, promoting a heterogenous primary nucleation pathway of αSyn aggregation. As the αSyn condensates aggregate at a faster rate with increasing amounts of Aβ40, they age faster, resulting in the condensates losing their liquid-like properties. Thus, they are unable to grow, ripen, or coalesce with other condensates. This explains why we observe a marked decrease in the average area of αSyn condensates as the concentration of Aβ40 was increased (Fig. 5*D*), as well as an increase in the overall number of condensates (Fig. 5*E*). Conversely, this behavior was seen to a much lesser extent when Aβ42 was mixed with αSyn. This is likely due to Aβ42 being much more aggregation-prone than Aβ40. Indeed, the scientific literature encompassing the kinetics of Aβ aggregation reports that the combined rate constants for Aβ42 aggregation are an order of magnitude larger than that of the Aβ40 peptide (41). Therefore, we found that, when Aβ42 at 5 μM and above was incubated with αSyn, Aβ42 first aggregated within the dilute phase prior to the onset of αSyn liquid–liquid phase separation. Remarkably, this gives rise to the ability of Aβ42 aggregates to nucleate the condensation of αSyn via liquid–liquid phase separation by acting as an anchor point (Fig. 4 *A* and *B*). In contrast, this behavior was not observed for Aβ40.

In the previous experiments, we used αSyn in excess compared to the concentration of Aβ42 and Aβ40. To further explore the effect of Aβ on αSyn condensation, we also investigated the possibility of αSyn and Aβ interacting at equimolar concentrations (*SI Appendix*, Fig. S3). For this, we mixed 100 µM αSyn with either 100 µM Aβ42 or 100 µM Aβ40. We found that, at such an elevated concentration, both Aβ42 and Aβ40 initially formed aggregates as visualized by ThT. Subsequently, αSyn monomers can be observed adsorbing to the surfaces of the Aβ aggregates. However, in contrast to the previous observations at lower Aβ42 concentrations, these aggregates did not nucleate the formation of liquid αSyn condensates, and αSyn liquid–liquid phase separation no longer occurred. This phenomenon can be attributed to the large amounts of Aβ aggregates present in the reaction mixture, sequestering the αSyn monomers. Thereby, free αSyn monomers are no longer available in sufficient quantities to form liquid condensates.

**Fig. 6.**
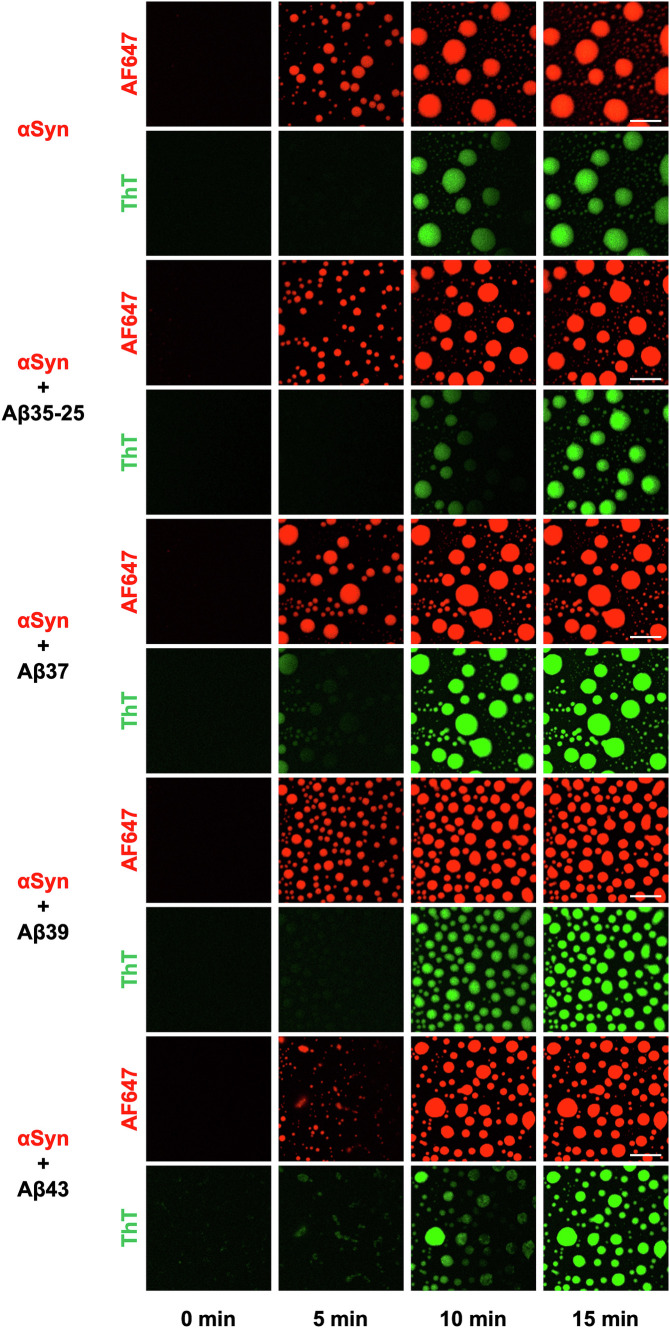
The effect of alternative Aβ variants on the condensation of αSyn. Confocal microscopy images of 100 µM αSyn (1% labeled with AF647) mixed with 20 µM of the indicated Aβ variants. The ThT channel displays the subsequent aggregation which takes place within the condensates. (Scale bar, 10 μm.)

Given the stark differences between Aβ42 and Aβ40 in their ability to modulate distinct steps in the phase transitions of αSyn, we expanded our study by testing alternative Aβ isoforms to gain a deeper understanding of the biophysical rules governing the interaction of Aβ with αSyn in the condensation pathway. To this end, we used the Aβ35-25 fragment, as well as the Aβ37 and Aβ39 variants, which are physiological cleavage products of Aβ40 and Aβ42, respectively, in addition to the longer Aβ43 variant. Furthermore, we assessed the effect of these variants on the aggregation of αSyn condensates using ThT (Fig. 6). We observed that, similar to Aβ40, Aβ35-25, Aβ37, and Aβ39 at 20 µM did not form any aggregates by themselves in solution and modulated the size and number of αSyn condensates (Fig. 6 and *SI Appendix*, Fig. S4). However, unlike Aβ40, these variants did not significantly affect the aggregation of αSyn within condensates, which may be related to their enhanced solubility and thus decreased propensity to form nuclei for αSyn aggregation, compared to Aβ40. In contrast, Aβ43 acted very much like Aβ42 and rapidly formed aggregates in solution before αSyn condensates appeared. The aggregates then served as nucleation sites for αSyn condensate formation. Based on these results, we conclude that Aβ variants can fall under one of two possible regimes with regard to their effect on αSyn condensation. Shorter, more soluble variants resemble the characteristics of the Aβ40 peptide, whereas longer variants like Aβ43 exhibit a similar behavior to Aβ42.

Finally, we verified whether the effects of Aβ42 and Aβ40 on αSyn phase separation were reproducible when mixed with a less condensation-prone variant of αSyn. For this, we chose αSyn-112, which lacks the C-terminal exon 5 of the αSyn protein sequence rendering it less prone to undergoing liquid–liquid phase separation and subsequent aggregation (29). Using this system, we were able to demonstrate that the addition of Aβ42 and Aβ40 also has profound effects on the phase separation behavior of αSyn-112 (*SI Appendix*, Fig. S5*A*). Both Aβ variants led to a strong decrease in the sizes and a concomitant increase in the number of αSyn-112 condensates (*SI Appendix*, Fig. S5 *B* and *C*). These results are in line with the observations for mixtures of Aβ42 and Aβ40 with full-length αSyn (Figs. 3 and 5) and thus corroborate the synergies between Aβ and αSyn reported in this work.

## Discussion

**Fig. 7.**
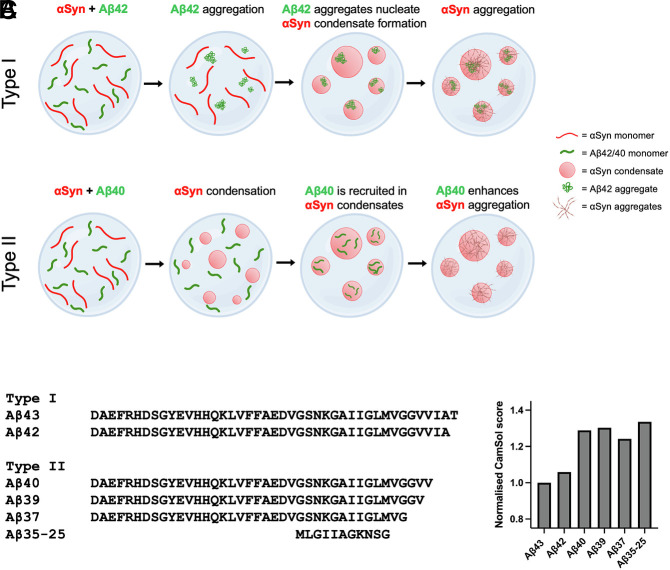
Kinetic diversity in the cocondensation of α-synuclein with amyloid-β variants. Aβ42 and Aβ40 exhibit different cocondensation behavior with αSyn. (*A*) Aβ42 undergoes nucleation events to form aggregates that initiate the nucleation of αSyn condensates (Type I). (*B*) Aβ40 remains soluble in its monomeric form and is recruited into condensates formed by αSyn (Type II). (*C*) Alternative Aβ variants follow one of the two pathways. Aβ43 forms aggregates that initiate the nucleation of αSyn condensates, as observed for Aβ42 (Type I). In contrast, Aβ37, Aβ39, and Aβ35-25 remain soluble in their monomeric form and are recruited into αSyn condensates, similar to Aβ40 (Type II). The behavior of the Aβ variants tested correlates with their CamSol scores, whereby variants with lower solubility values (Aβ43 and Aβ42) are prone to forming aggregates themselves, whereas variants with higher solubility values (Aβ40, Aβ39, Aβ37, and Aβ35-25) remain soluble at first.

In this work, we have probed how Aβ variants form cocondensates with αSyn through different kinetic mechanisms. Using a combination of biophysical techniques, chemical kinetics, and fluorescence microscopy, we demonstrated that the two main variants of Aβ (Aβ40 and Aβ42) interact with αSyn in distinct manners. While monomeric Aβ40 is recruited into αSyn liquid condensates, Aβ42 forms aggregates prior to the onset of αSyn liquid–liquid phase separation. These Aβ42 aggregates subsequently act as nucleation sites, or anchor points, which promote the condensation of αSyn (Figs. 2, 4 and 7). Moreover, we have explored how additional variants of Aβ such as Aβ35-25, Aβ37, Aβ39, and Aβ43 interact with αSyn. We found that Aβ43 behaves much like Aβ42, first forming aggregates in solution, which then act as nucleation sites from which αSyn forms liquid condensates. In contrast, Aβ35-25, Aβ37, and Aβ39 behave similarly to Aβ40, as they remain soluble at first and interact with αSyn, modulating condensate size and number (Fig. 6 and *SI Appendix*, Fig. S4). This kinetic diversity provides insight into the complex pathways in which cocondensation may arise and provides an understanding of the intricate mechanisms through which Aβ and αSyn interact with one another.

Furthermore, we investigated the liquid-to-solid transition of the αSyn condensates when mixed with different Aβ variants. Our results suggest that two distinct mechanisms of action occur. In the first mode of action, we found that Aβ42 and Aβ43 themselves aggregate and are not involved in the aggregation of the αSyn condensates. In other words, Aβ42 and Aβ43 only influence the liquid–liquid phase separation of αSyn by acting as nucleation sites but have no effect on the liquid-to-solid transition. This is in contrast to Aβ40, which has the ability to enhance the aggregation of these condensates. However, the shorter variants Aβ37, Aβ39, and Aβ35-25 did not modulate the aggregation of αSyn condensates. This is likely due to the lack of the C-terminal amino acids of the Aβ sequence, which decreases the propensity of these isoforms to aggregate (41–43). These findings are in line with studies investigating the molecular interactions between αSyn and Aβ isoforms. For both, Aβ42 and Aβ40, the C-terminal region has been shown to be implicated in mediating the binding of αSyn. Moreover, under certain conditions, various regions of Aβ42, including the N-terminus and the central Aβ22-35 sequence, were found to be involved in the interaction with αSyn (44, 45). However, Aβ42 differs from Aβ40 only by the two hydrophobic C-terminal amino acids, isoleucine, and alanine. It is therefore quite plausible that the exact amino acid composition dictates which parts of the molecule engage with αSyn in the cocondensation and coaggregation process, which may be highly sensitive to the biophysical properties of each individual Aβ variant. Furthermore, it appears that these two hydrophobic amino acids are key to the different mechanisms exhibited by Aβ42 and Aβ40. This, however, is not particularly surprising as the aggregation of these two peptides has been shown to greatly vary (44, 45).

Interestingly, no condensate formation was observed when αSyn was incubated with Aβ42 or Aβ40 at equimolar ratios. Instead, due to the large Aβ concentration used (100 µM), both Aβ42 and Aβ40 aggregated almost instantaneously, and αSyn monomers were sequestered on the surface of the Aβ aggregates formed in situ. This observation is in agreement with reports indicating that Aβ aggregates can bind αSyn monomers, as well as other disordered proteins and peptides (46–48). Therefore, an important conclusion that arises is that Aβ concentration can determine whether αSyn will phase-separate or directly coaggregate.

Furthermore, we showed that the effect of Aβ42 and Aβ40 on αSyn condensate formation is preserved when using a less condensation-prone αSyn variant (*SI Appendix*, Fig. S5). The 112-residue splice isoform (αSyn-112) lacks the protein sequence encoded by exon 5, which is located in the flexible, negatively charged C-terminus of αSyn and was previously shown to enhance αSyn phase separation (17, 29). Mounting evidence from recent years reveals that changes in alternative splicing may play a crucial role in αSyn aggregation in synucleinopathies (15, 49, 50), and the disparate behavior between Aβ variants and αSyn phase separation which we report here further adds to the idea that protein complexity can have a massive influence on the diversity of the system. The mechanism of action regarding how different Aβ variants affect αSyn phase separation and aggregation is shown in Fig. 7. We found that all Aβ variants generally followed one of two possible pathways in the cocondensation with αSyn. The more aggregation-prone variants (Aβ43 and Aβ42) formed aggregates first, which then initiated the condensation of αSyn, whereas the less aggregation-prone variants (Aβ40, Aβ39, Aβ37, and Aβ35-25) remained soluble at first and were recruited into αSyn condensates. This behavior correlates with the CamSol solubility scores of the variants, with Aβ43 and Aβ42 having lower solubility scores than the other four variants (Fig. 7*C*).

Up to date, there are numerous studies showing that neuropathological comorbidities between αSyn and Aβ exist in various diseases. On the one hand, the NAC sequence was initially identified in amyloid plaques in AD before αSyn was identified as a major component of Lewy bodies (7). In the following years, an increasing body of literature has demonstrated the presence of Aβ deposits in synucleinopathies (51–54) and, conversely, αSyn deposits in AD (55–58). In line with this, a synergy between the two types of lesions in the disease process has been put forward (52, 53).

Moreover, it should be noted that, in cellular environments, there are several mechanisms which could elicit the interaction of αSyn and Aβ, which are usually located in the cytosol and extracellular space, respectively. However, under pathological conditions, both proteins have been reported to be present in various cellular compartments such as endosomes, autophagosomes, lysosomes, and mitochondria (59). Multiple studies have shown that Aβ may accumulate intracellularly, where it is resistant to degradation (60, 61). First, extracellular Aβ peptides can be internalized by endocytosis and thereby enter the endolysosomal pathway (31–33). In support of this, labeled Aβ peptides, when added externally to cultured cells at nanomolar concentrations, are rapidly taken up via endocytosis and reach lysosomes where they accumulate. This process enhances the effective concentration of Aβ into the micromolar range and thereby facilitates Aβ aggregation (31, 32). Moreover, failure of the lysosomal pathway and lysosome leakage may allow Aβ peptides to escape into the cytoplasm where αSyn is mainly located (62, 63). Conversely, αSyn may reach lysosomes via macroautophagy or in exosomes entering neighboring cells. Finally, cells may release αSyn into the extracellular space, via exocytosis or due to cellular disintegration (64, 65), where αSyn can come into contact with Aβ. Therefore, advancing our fundamental knowledge in this area is of key importance if we are to understand the interactions between these proteins. Moreover, exploring the role of Aβ and αSyn in the cellular processes underlying neurodegenerative disorders will be pivotal in targeting the correct mechanistic steps with the goal of developing effective, disease-modifying therapeutics. In fact, if we are to find appropriate pathways at targeting neurodegeneration and provide efficient therapeutic avenues, we must first understand the root-causes of the disease and the fundamental protein interactions that occur at the molecular level.

These results illustrate the importance of investigating complex systems composed of different proteins, where interactions between proteins can lead to cocondensation and coaggregation phenomena. Importantly, we demonstrated the kinetic diversity that exists in the phase behavior between variants of Aβ and αSyn. Through such approaches, a better understanding of the molecular mechanisms which govern protein phase separation and subsequent aggregation in the context of neurodegenerative diseases can be elucidated. This knowledge can in turn facilitate the identification of pharmacological molecules that specifically target such interactions.

## Materials and Methods

### Purification and Labeling of αSyn.

Human full-length αSyn, αSyn-112, and their respective cysteine variants (A90C) were expressed in BL21 Gold (DE3) competent cells transformed with a pT7-7 plasmid (αSyn and αSyn-A90C) or a pET29a(+) plasmid (αSyn-112, αSyn-112-A90C). αSyn was subsequently purified in 50 mM trisaminomethane-hydrochloride (Tris-HCl) at pH 7.4 based on previously established protocols (15). The A90C variant of αSyn was labeled with an excess of Alexa Fluor 647 C2 Maleimide (AF647, Invitrogen Life Technologies) overnight at 4 °C with continuous inversion. Any unbound dye was removed using Amicon Ultra-15 Centrifugal Filter Units and buffer exchanged into 50 mM Tris-HCl, pH 7.4. The A90C variant of αSyn-112 was labeled with Alexa Fluor 555 C2 Maleimide in the same way. The final labeled protein concentration was determined from the Beer–Lambert law by measuring the UV/VIS absorbance using the extinction coefficient ε = 239,000 M^−1^ cm^−1^ for AF647 and ε = 155,000 M^−1^ cm^−1^ for AF555, respectively.

### Purification and Labeling of Aβ42, Aβ40, and Further Aβ Variants.

Human wild-type Aβ42/40 and the Aβ42 cysteine variant (cysteine insertion at amino acid position 2) were expressed in BL21 Gold (DE3) competent cells, transformed with a pT7 plasmid encoding the relevant Aβ variant. The Aβ variants were subsequently purified in 50 mM Tris-HCl, pH 7.4, using previously established protocols (66, 67). All Aβ was aliquoted, flash-frozen in liquid nitrogen, lyophilized, and stored at −80 °C. Lyophilized Aβ was resuspended in 50 mM Tris-HCl, pH 7.4, on ice before each experiment. The cysteine-bearing Aβ42 variant was independently resuspended in 50 mM sodium phosphate buffer, pH 7.5, labeled with an excess of Alexa Fluor 488 C5 Maleimide (AF488, Invitrogen Life Technologies) and incubated at room temperature for 2 h. Labeled Aβ was separated from unbound dye and Aβ dimers by size exclusion chromatography (Superdex 75 10/300 GL column) using 50 mM Tris-HCl, pH 7.4, at a flow rate of 0.7 mL/min. The concentration of labeled Aβ42 was determined from the Beer–Lambert law by measuring the UV/VIS absorbance using the extinction coefficient ε = 73,000 M^−1^ cm^−1^ for AF488. Lyophilized peptide stocks of Aβ35-25, Aβ37, Aβ39, Aβ43 were obtained from Bachem AG. Peptides were resuspended in 50 mM Tris-HCl and immediately used for liquid–liquid phase separation experiments. Solubility scores were calculated using CamSol-PTM at pH 7.4 (68).

### Liquid–Liquid Phase Separation Assay.

The liquid–liquid phase separation assay utilized in this work has been taken from a previously established protocol (23). In short, a sample mixture was prepared in 50 mM Tris-HCl, pH 7.4, consisting of 100 μM αSyn, with varying concentrations of Aβ, and 5% (w/w) polyethylene glycol (PEG). Then, 10 μL of the sample was pipetted using a drop-casting approach onto a glass-bottom dish. The sample was immediately imaged using confocal microscopy (Leica Stellaris 5). Excitation wavelengths were set at 650 nm for αSyn (1% AF647-labeled) and 490 nm for Αβ42 (10% AF488-labeled). All images were processed and analyzed using Fiji (69).

### Protein Aggregation Assay within Liquid Condensates.

To study the aggregation of proteins within condensates, the sample mixture outlined above was supplemented with 20 μM thioflavin T (ThT). Only wild-type Αβ was used to eliminate potential overlaps between the fluorescently labeled protein and ThT. Then, 10 μL of the sample was drop-cast onto a glass-bottom dish and was imaged using confocal microscopy (Leica Stellaris 5) at excitation wavelengths 650 nm αSyn and 405 nm for ThT. The ThT fluorescence intensity was extracted from time-lapse images analyzed using Fiji (69).

### Statistical Analysis.

All statistical analyses were performed in GraphPad Prism 9 (GraphPad Software). Data are presented as the mean with standard error of the mean from at least three independent biological replicates, unless indicated otherwise.

## Supplementary Material

Appendix 01 (PDF)

Movie S1. Video showing the formation of Aβ42 aggregates, which in turn act as anchor points through which αSyn phase separates.

Movie S2. An example of Aβ42 aggregates nucleating the condensation of αSyn via liquid-liquid phase separation, and the subsequent liquid-to-solid transition of αSyn.

Movie S3. Another example of Aβ42 aggregates nucleating the condensation of αSyn via liquid-liquid phase separation, and the subsequent liquid-to-solid transition of αSyn.

## Data Availability

All study data are included in the article and/or supporting information.
