# Supplementary material for: Amyloid-β modulates the phase separation and aggregation of α-synuclein
Source: Proc Natl Acad Sci U S A. 2025 Jul 14;122(29):e2501987122. doi: 10.1073/pnas.2501987122 (PMC12305028; doi:10.1073/pnas.2501987122)
Supplement: Supplementary file 1 — Appendix 01 (PDF) [file pnas.2501987122.sapp.pdf]

SUPPORTING INFORMATION

Amyloid-β modulates the phase separation and aggregation of α-synuclein

Alexander Röntgen[+], Zenon Toprakcioglu[+*], Owen M. Morris[+], Michele Vendruscolo[*]

Centre for Misfolding Diseases, Yusuf Hamied Department of Chemistry, University of Cambridge, Cambridge CB2 1EW, UK

+ *Equal contributions*
* *Correspondence to: zt231@cam.ac.uk, mv245@cam.ac.uk*

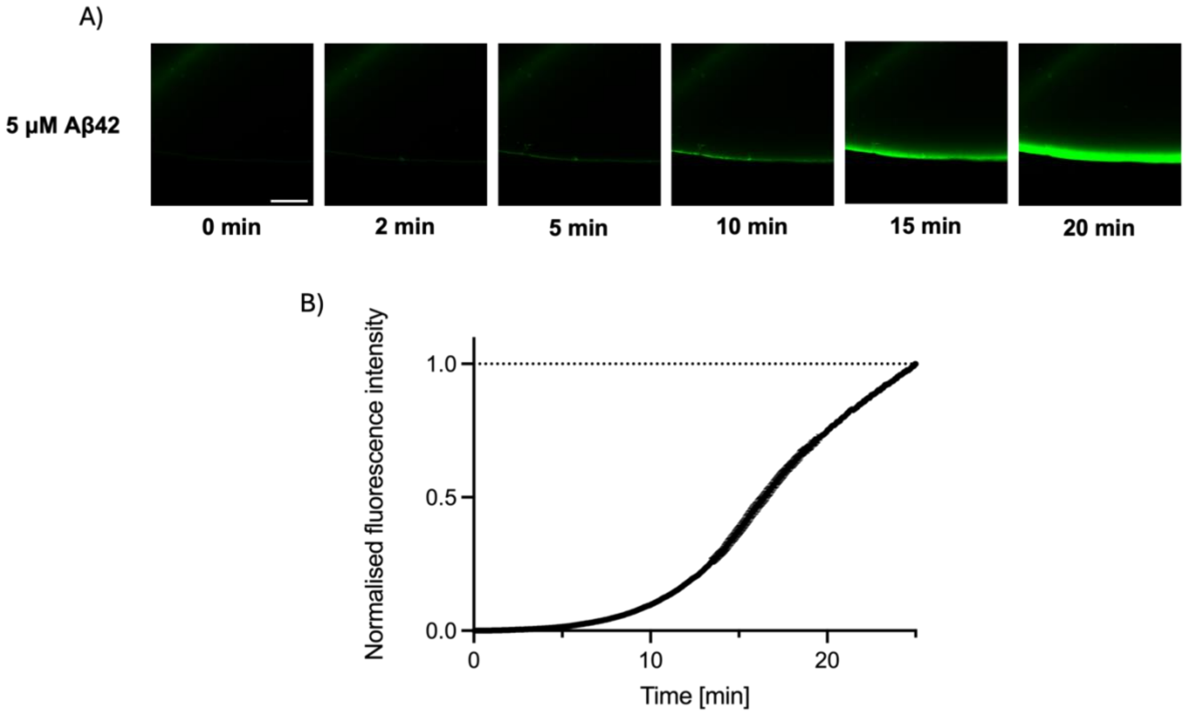

Supplementary Figure 1. Aβ42 aggregation in the absence of αSyn. (A) Time-lapse confocal microscopy images of 5 µM Aβ42 with 5% (w/w) PEG. Under these conditions, in the absence of αSyn, Aβ42 does not undergo liquid-liquid phase separation. The ThT channel indicates that Aβ42 does not undergo aggregation via a liquid condensation pathway. Scale bar = 10 µm. **(B)** ThT kinetic aggregation data indicating Aβ42 aggregation in a dilute phase when 10 µL sample droplet is suspended onto a microscope slide.

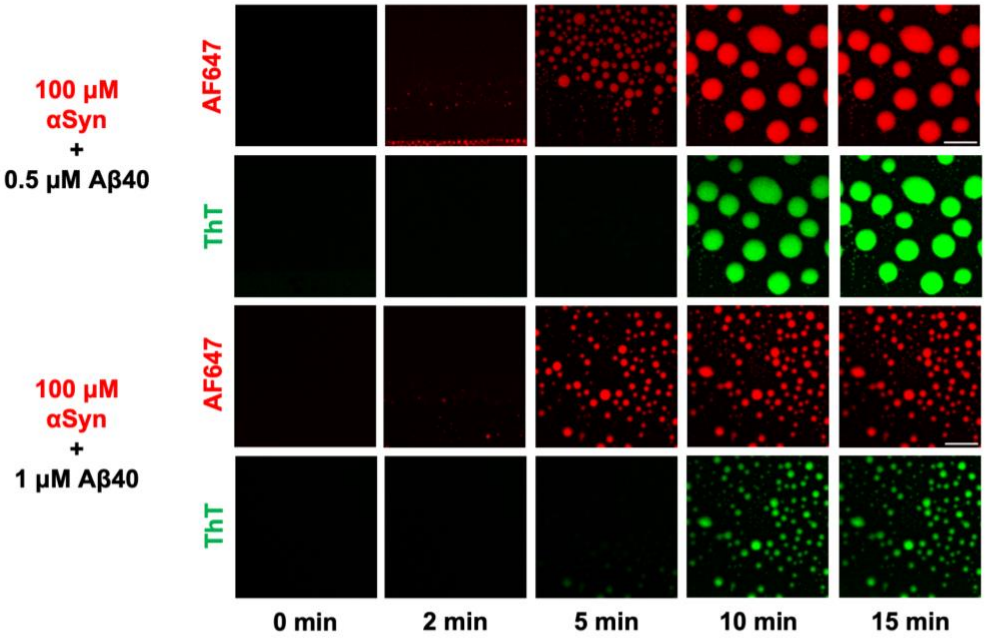

Supplementary Figure 2. αSyn condensation in the presence of different concentrations of Aβ40. Time-lapse confocal microscopy images of αSyn condensates (1% labelled with AF647) with Aβ40. The ThT channel displays the subsequent aggregation which takes place within the condensates. Scale bar = 10 µm.

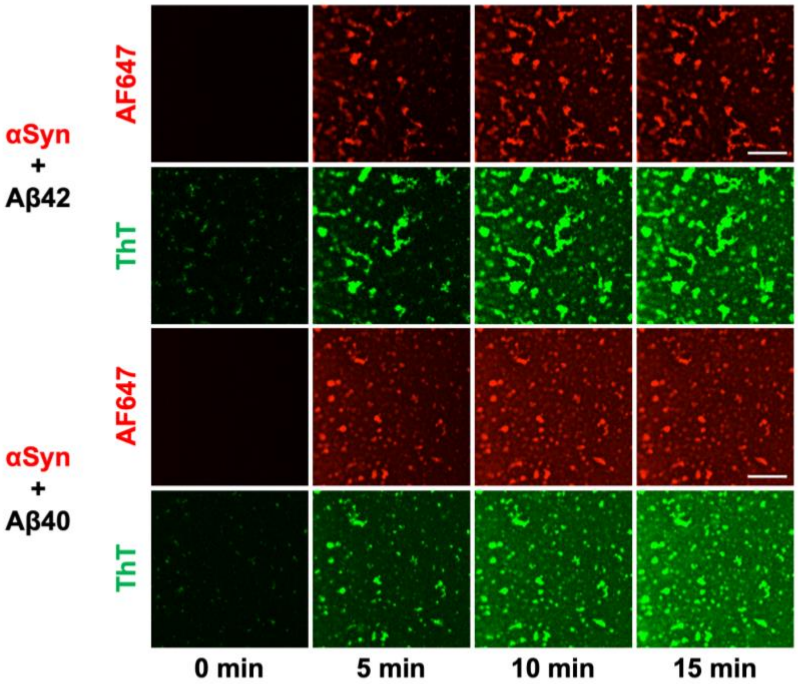

Supplementary Figure 3. αSyn condensation with equimolar concentrations of Aβ. Time-lapse confocal microscopy images of 100 μM αSyn (1% labelled with AF647) with 100 μM Aβ. The ThT channel displays the initial aggregation of Aβ, and the subsequent aggregation of αSyn which binds to the Aβ aggregates. Scale bar = 10 μm.

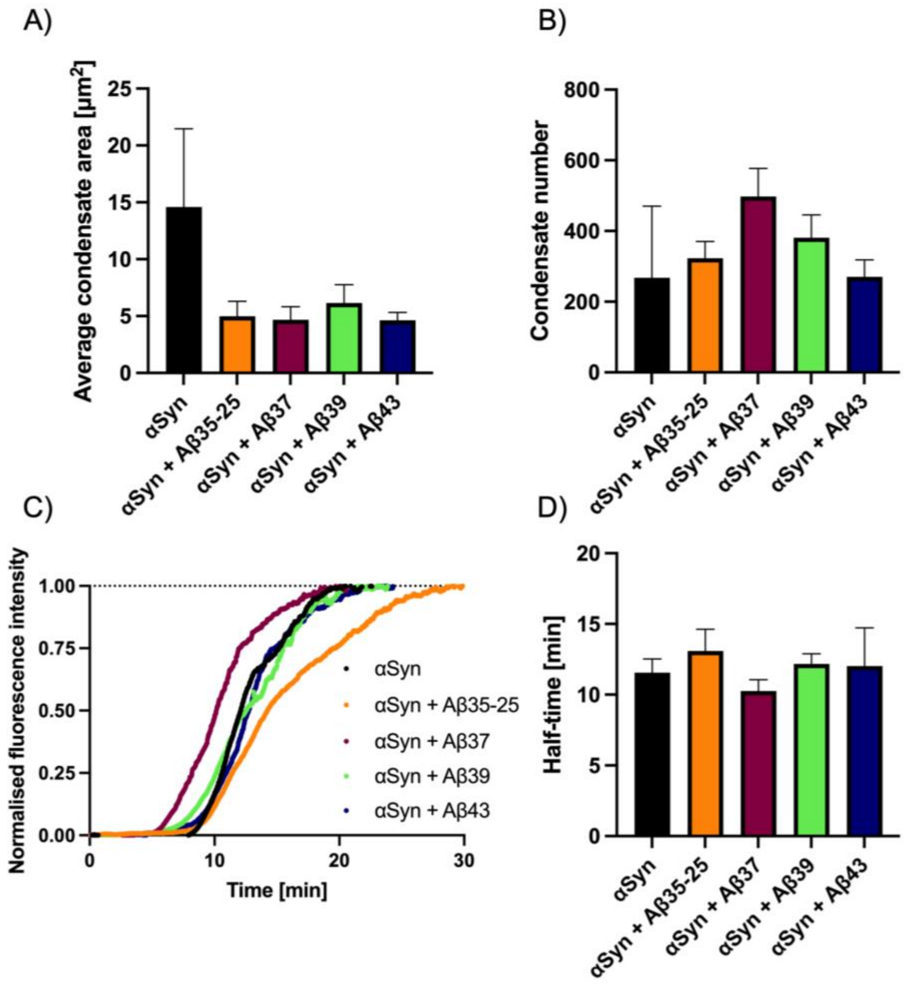

Supplementary Figure 4. Biophysical analysis of the effect of alternative Aβ variants on the condensation of αSyn. (A,B) Plots showing (A) the average area and (B) the number of αSyn condensates at 15 min (shown in Figure 6). **(C)** Normalised median kinetic traces corresponding to the aggregation of αSyn within condensates in the presence of alternative Aβ variants. **(D)** Half-time plots of the corresponding kinetic traces from (C).

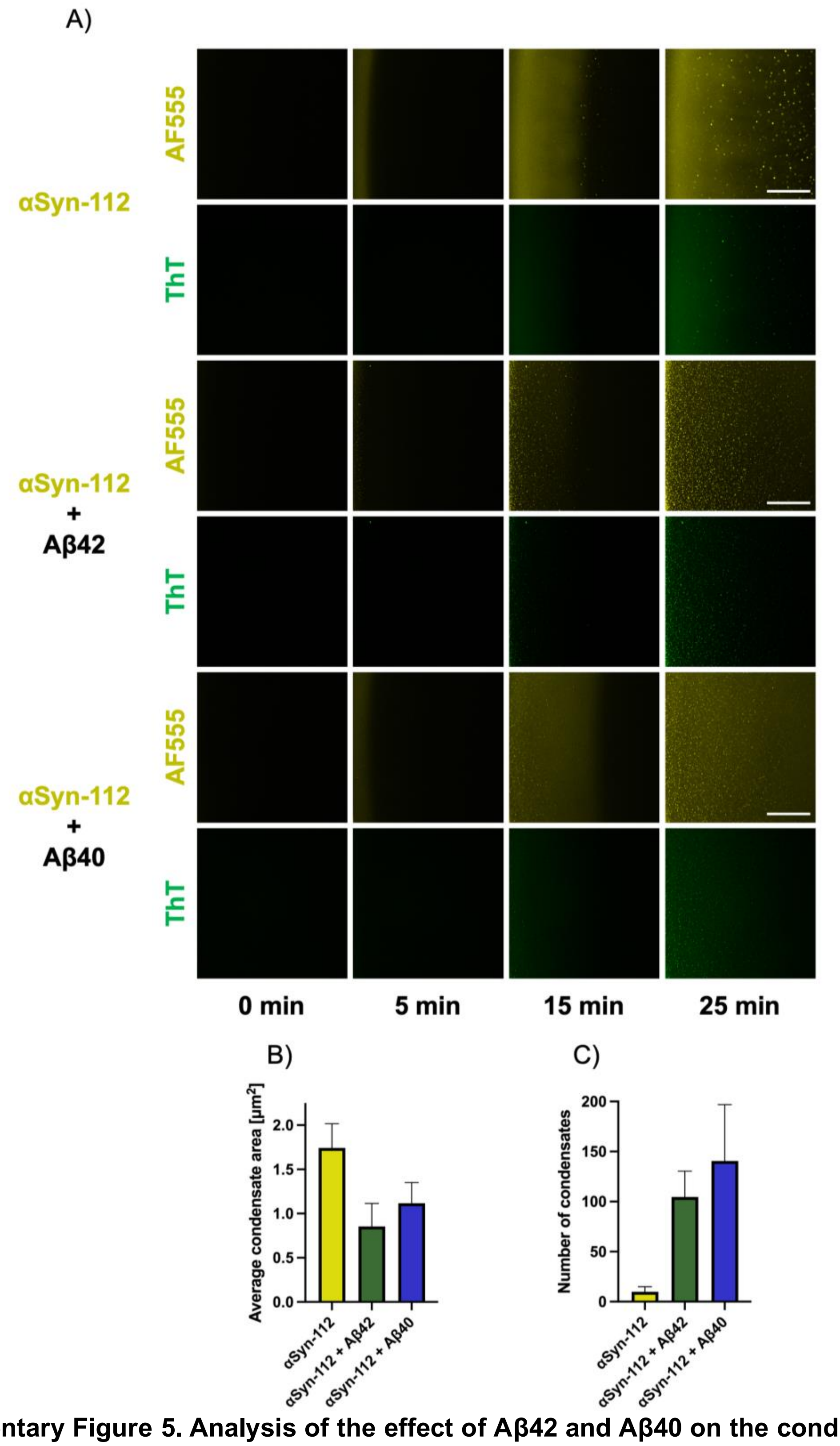

Supplementary Figure 5. Analysis of the effect of Aβ42 and Aβ40 on the condensation of the alternative splice isoform αSyn-112. (A) Confocal microscopy images of 100 µM αSyn-112 (1% labelled with AF555) mixed with 20 µM of Aβ42 or Aβ40. The ThT channel displays the subsequent aggregation which takes place within the condensates. Scale bar = 100 µm. **(B)** Plot showing the average area of αSyn-112 condensates at 25 min. **(C)** Plot of the number of αSyn condensates per 10^4 μm^2 at 25 min.

Movie S1. Video showing the formation of Aβ42 aggregates, which in turn act as anchor points through which αSyn phase separates.

Movie S2. An example of Aβ42 aggregates nucleating the condensation of αSyn via liquid-liquid phase separation, and the subsequent liquid-to-solid transition of αSyn.

Movie S3. Another example of Aβ42 aggregates nucleating the condensation of αSyn via liquid-liquid phase separation, and the subsequent liquid-to-solid transition of αSyn.
